# Secure and efficient implementation of facial emotion detection for smart patient monitoring system

**DOI:** 10.15302/J-QB-022-0312

**Published:** 2023-06-01

**Authors:** Kh Shahriya Zaman, Md Mamun Bin Ibne Reaz

**Affiliations:** ^1^ Department of Electrical Electronic and Systems Engineering Universiti Kebangsaan Malaysia (UKM) Bangi 43600 Malaysia

**Keywords:** facial expression detection, emotion recognition, FPGA implementation, convolutional neural network, signed digit approximation

## Abstract

**Background:**

Machine learning has enabled the automatic detection of facial expressions, which is particularly beneficial in smart monitoring and understanding the mental state of medical and psychological patients. Most algorithms that attain high emotion classification accuracy require extensive computational resources, which either require bulky and inefficient devices or require the sensor data to be processed on cloud servers. However, there is always the risk of privacy invasion, data misuse, and data manipulation when the raw images are transferred to cloud servers for processing facical emotion recognition (FER) data. One possible solution to this problem is to minimize the movement of such private data.

**Methods:**

In this research, we propose an efficient implementation of a convolutional neural network (CNN) based algorithm for on‐device FER on a low‐power field programmable gate array (FPGA) platform. This is done by encoding the CNN weights to approximated signed digits, which reduces the number of partial sums to be computed for multiply‐accumulate (MAC) operations. This is advantageous for portable devices that lack full‐fledged resource‐intensive multipliers.

**Results:**

We applied our approximation method on MobileNet‐v2 and ResNet18 models, which were pretrained with the FER2013 dataset. Our implementations and simulations reduce the FPGA resource requirement by at least 22% compared to models with integer weight, with negligible loss in classification accuracy.

**Conclusions:**

The outcome of this research will help in the development of secure and low‐power systems for FER and other biomedical applications. The approximation methods used in this research can also be extended to other image‐based biomedical research fields.

## Abstract 2

**Author summary:** With the advent of the Internet of Things and Big Data analysis, it is possible to create an intelligent ecosystem for continuous patient monitoring with minimal human interaction. But the machine learning algorithms associated with such smart healthcare ecosystems are usually resource‐intensive and require communication of private data to cloud servers. In this article, we proposed an approximation method for neural networks, which can be efficiently implemented on FPGAs, and therefore enable on‐device FER. We implemented several CNN models on the FPGA for FER. Our approximated method can save at least 22% FPGA resource usage, which is advantageous for embedded systems.

## INTRODUCTION

The growing role of artificial intelligence (AI) in medicine and healthcare is on the cusp of changing the medical profession forever. The large availability of biomedical data brings tremendous opportunities and challenges to health care research. Several studies demonstrated the potential of deep learning to replace tasks that currently require highly trained physicians, such as reading CT scans of the head, detecting breast cancer from thermography, screening for disease from chest X‐rays, diagnosis of diabetes, and many more [1, 2, 3, 4, 5, 6]. Such diagnostic medical specialities rely on the interpretation of data, rather than direct patient interactions. Therefore, these AI technologies are augmenting doctors and medical professionals with faster and more accurate decision‐making capabilities. Furthermore, an AI‐based continuous monitoring system allows medical professionals to remotely access real‐time data, which is beneficial when dealing with highly contagious diseases or isolated intensive care units [7]. AI in the critical care setting can reduce nurses’ workload to allow them to spend time on more critical tasks, thus enabling better‐personalized care through low‐cost and high‐capacity intelligent data processing.

Facial emotions are one of the most important parts of the non‐verbal communication array that humans possess. Several researchers suggested that facial expressions of emotions are used as signals of health status [8, 9, 10]. The identification and correct interpretation of facial expressions play an important role in the comfort level or non‐verbal reaction to the medical treatment of a patient. This helps the doctor understand and respond to the deviations from the normal facial expression spectrum that are present in their patients, especially those who suffer from physical or emotional pain.

Facial expression for emotion detection has always been an easy task for humans, but achieving the same task with a computer algorithm is quite challenging. Most techniques process visual data and search for general patterns present in images or videos of human faces. Some research even utilized multimodal data, containing electroencephalogram (EEG) data, speech data, text data, 3D facial data, or thermal data [11, 12, 13, 14]. Although geometrical features of the 3D facial model are effective for facial emotion recognition (FER), they fail to detect subtler characteristics like skin texture changes, as 3D data does not convey appearance information. Appearance features from 2D images are more stable to noise, allowing for the detection of a more complete set of features, being particularly important for detecting micro‐expressions. These feature extraction techniques can be applied to both RGB and thermal modalities [15]. Furthermore, a survey on emotion detection methods concluded that the emotion detection accuracy gained from multimodal systems is not worth the additional complexity and computational resources [16]. This is more prevalent in the case of embedded systems with limited resources. As for EEG data, its use for patient monitoring is unfavourable due to its invasive nature, which might cause discomfort to the patients.

Compared to conventional feature extraction techniques, deep learning (DL) based algorithms, especially convolutional neural networks (CNN), have been shown to perform consistently better on different facial emotion detection datasets [17,18]. CNNs can automatically extract the features and learn the pattern for different emotion classes. However, CNNs often require substantially more computational resources than conventional methods. This limits their implementation on low‐power portable devices.

A CNN model requires numerous matrix multiplications, which are carried out by the multiply‐accumulate (MAC) units of the hardware. These calculations are generally carried out by floating‐point multipliers, which consume more power and area than integer multipliers. By leveraging the fact that CNNs are tolerant to low‐precision computation [19, 20, 21], different forms of approximation can be applied to reduce computational requirements.

The number of nonzero digits in a weight representation is directly related to the number of partial sums to be computed in a MAC operation. One way to reduce the number of nonzero digits is to adopt the signed digit representation. Minimum signed digit (MSD) [22,23] representation is another ternary system, that ensures fewer nonzero digits than its binary equivalent. As a result, the number of partial sums to be computed is reduced, which substantially reduces the computations in MAC operations for the overall network. This is also beneficial for embedded processors that lack fully combinational multipliers and rely on adders for MAC operations.

### Related research

There have been many attempts to make an automatic facial expression analysis tool as it has applications in many fields such as robotics, medicine, driving assist systems, and lie detector [17,18,24]. Most conventional approaches for FER primarily focus on facial investigation keeping background intact and hence building up a lot of unnecessary and misleading features that confuse the training process. In such cases, the training and testing dataset are pre‐processed to only contain the facial image.

For embedded applications, it is desirable to work with simple datasets with basic classifiable emotions. The Facial Expression Recognition 2013 dataset (FER2013) [25], the Japanese Female Facial Expression (JAFFE) dataset [26], and the Karolinska Directed Emotional Face dataset (KDEF) [27] are image datasets containing seven different classes of emotions, namely angry, disgust, fear, happy, neutral, sad and surprise. The FER2013 dataset is particularly suitable for low‐resource computation, as it contains grayscale images at a resolution of 48 by 48 pixels. The JAFFE and KDFE datasets contain images with much higher resolutions. Despite the low‐resolution images, most CNN models can attain respectable classification accuracy for emotion detection with the FER2013 dataset.

The MobileNet‐v2 [28] is a CNN architecture developed by Google for embedded image classification applications. MobileNet‐v2 can achieve relatively high classification accuracy (68% to 72%) considering with its small model size containing 2.3 million parameters [29,30]. Some researchers have successfully implemented a MobileNet‐v2 model on field programmable gate array (FPGA) by quantizing the weight parameters of a pretrained model for FER. The floating‐point weights were converted to fixed‐point representations, thereby effectively replacing all floating‐point multipliers with FPGA resource‐friendly integer multipliers [31].

The residual neural network (ResNet) [32] is another popular CNN that utilizes skip connections to attain state‐of‐the‐art classification accuracies on most image analysis tasks. ResNet18 is one of the small variants of the ResNet family. However, it still contains around 11 million parameters. As a result, it can achieve up to 5% better accuracy than MobileNet‐v2 on the same FER2013 dataset. Although this model consumes more FPGA resources, it is less likely to suffer from quantization errors on FPGA [33]. Thus, it is suitable for portable applications where higher classification accuracy is a priority.

Other than the commonly used CNN architectures, some researchers designed their own lightweight CNN models containing fewer parameters and neuron connections to simplify the CNN model and reduce FPGA resource usage. Such handcrafted models consumed up to 45% fewer FPGA resources, but the classification accuracy achieved was 60% at best [34,35].

### Contributions

In this article, we propose a weight approximation scheme for the efficient implementation of CNNs on low‐power ASICs and FPGAs. The CNN models are pretrained on the FER2013 dataset [24], which contains facial images of seven different classes of emotions. The contributions of this article can be summarized as follows:

• A minimum signed digits (MSD) based approximation method is proposed that reduces the computation complexity of CNN‐based FER models. The approximation reduces the number of nonzero digits in MSD representation for the weights in the pretrained CNN model. This helps in lowering the number of partial products to be computed for multiplication operations.

• The proposed method is demonstrated with MobileNet‐v2 on FPGA and ResNet18 models through simulations, which are pretrained with the FER2013 dataset.

• Based on the results of our experiments, some strategies for applying the proposed approximation scheme are provided for secure and on‐device FER, and some future research directions are outlined in this area.

## RESULTS AND DISCUSSIONS

To analyze the effectiveness of the proposed approximation method, a Python script was used to count and compare the number of nonzero digits in the first convolution layer of MobileNet‐v2. This layer contains a total of 3 × 3 × 64 = 576 parameters. The results are shown in Tab.1.

The total number of nonzero digits used for generating partial sums drops quite significantly when the parameters are approximated with MSD with *p* = 1, 2, and 3. It should be noted that only the nonzero bits in the mantissa group for floating‐point weights were considered for comparison, as only these 23 bits contributeto multiplication operations. Using only three nonzero digits to represent each weight reduces the total number of shift‐and‐add operations by almost 85% in the first convolution layer. We can expect similar results for the rest of the parameters in the MobileNet‐v2 model. The nonzero digits can be further reduced with *p* = 2 and 1. However, the model classification accuracy is likely to suffer from this.

**Table 1:** Analysis of nonzero digits in the weights of the first convolutional layer of MobileNet‐v2

Weight representation	32‐bit floating‐point	16‐bit approximated MSD		
		*p*=3	*p*=2	*p*=1
Number of nonzero digits	7677*	1159	978	538

* Only mantissa bits were considered

To multiply the approximated MSD weights with binary activations, the custom multiplier was designed to operate as a shift‐and‐add MAC unit. Such designs use fewer FPGA resources than a floating‐point and a fully combinational 16‐bit multiplier. We compared the implementation area and latency trade‐offs of the custom MAC designs with the open‐source implementations of commonly used exact multipliers. Their synthesis results along with normalized mean error distance (NMED) from the approximation of weights are reported in Tab.2. The errors were estimated from extensive simulations from all possible 16‐bit input values to the integer multipliers. The NMED value was calculated from the mean of all absolute errors divided by the maximum output of the multiplier. The custom MAC units require fewer FPGA resources compared to conventional exact multipliers. Compared to the floating‐point multipliers present in most general‐purpose CPUs, the custom multipliers are at least 17× more resource‐efficient and 1.4× faster.

The FPGA implementation results of the FER model based on MobileNet‐v2 with approximated MSD weights are shown in Tab.3. The table also shows the classification accuracy for ResNet18 models for different values of *p*. The onboard DSPs were disabled for a fair comparison. As a baseline, a MobileNet‐v2 model with 8‐bit integer quantized weights was implemented. Although the lower bit‐width saved almost 50% memory compared to 16‐bit MSD approximated weights models, there was a drop in accuracy as classification accuracy and the latency for each MAC operation was higher for integer multipliers. The overall FER model with *p* = 3 requires almost 22% fewer FPGA resources than the integer model. The smallest model with *p* = 1 can operate at a higher clock speed since almost all the MAC operations are essentially replaced by a single shift‐and‐add operation.

**Table 2:** Synthesis results for different multipliers designs

Multiplier	Total logic elements	Latency	NMED
Booth 8‐bit	257	25.27 ns	−
Booth 16‐bit	907	28.33 ns	−
FP32	1068	36.38 ns	−
MSD‐MAC,*p=3*	61	20.87 ns	0.2
MSD‐MAC,*p=2*	26	18.26 ns	1.8
MSD‐MAC, *p=1*	3	11.25 ns	10.6

**Figure 1:**
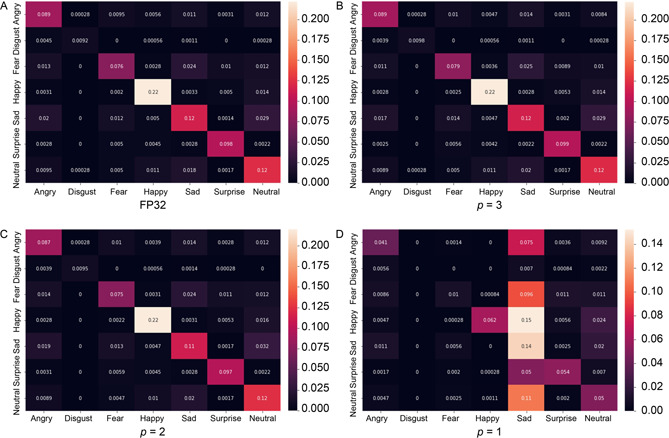
Confusion matrix for classification performance on the FER2013 dataset by MobileNet‐v2 models with (A) floating‐point weights (FP32), MSD approximated weights with (B) *p* = 3, (C) *p* = 2 and (D) *p* = 1.

The classification performance of the MobileNet‐v2 and ResNet18‐based FER models is summarized in Tab.4. The ResNet18 models were not implemented on hardware due to the memory limitations of the FPGA board. The results are collected through software simulations. Compared to MobileNet‐v2 models, ResNet18 retains better accuracy at lower precision weights. Therefore, ResNet18 models can be deployed with *p* = 2 approximation for higher model accuracy and faster inference, but at the expense of high lookup table (LUT) usage. On the other hand, MobileNet‐v2 models are relatively smaller and therefore require fewer FPGA resources. However, these models are less tolerant to high approximation errors of weights. These results are inherent in the confusion matrix for FER using different variants of MobileNet‐v2 in Fig.1.

**Table 3:** Synthesis results for MobileNet‐v2 models with different levels of approximations

FER model	LUT usage	BRAM	Fmax
MobileNet‐v2 (int8)	258,648	13,001 Kb	120 MHz
MobileNet‐v2 (*p*=3)	202,482	26,002 Kb	155 MHz
MobileNet‐v2 (*p*=2)	196,587	26,002 Kb	155 MHz
MobileNet‐v2 (*p*=1)	171,549	26,002 Kb	170 MHz

**Table 4:** FER Accuracy of different MobileNet‐v2 models with FPGA simulation

FER model		Top 1 accuracy	Top 2 accuracy	Loss	Precision
MobileNet‐v2	FP32	71.3%	82.1%	0.016	0.714
	*p*=3	70.5%	80.9%	0.016	0.692
	*p*=2	68.8%	79.0%	0.017	0.689
	*p*=1	36.0%	57.9%	0.026	0.360
ResNet‐18	FP32	73.7%	86.0%	0.014	0.737
	*p*=3	73.3%	85.3%	0.015	0.733
	*p*=2	72.4%	84.7%	0.015	0.725
	*p*=1	46.1%	69.0%	0.023	0.461

## CONCLUSION

In this paper, we demonstrated the use of approximated minimum signed digit encoding for efficient implementation of on‐device facial emotion recognition. Specifically, we reduced the number of partial sum computations by reducing the number of nonzero digits in the weight parameters of the FER model. The technique was accompanied by a custom MAC unit for FPGAs to efficiently compute the activations from the approximated weights. The custom MAC units are at least 17× more resource‐efficient and 1.4× faster than commonly used floating‐point multipliers. This enables the implementation of the whole FER model on low‐resource hardware, using fewer resources compared to general‐purpose exact multiplier‐based models. This was achieved with almost no loss in emotion classification accuracy. Using the techniques demonstrated in this paper, it would be possible to deploy low‐power and portable emotion recognition devices, which will not rely on cloud servers for computations, and thereby the system would be more secure for smart monitoring systems from a privacy perspective of the patients.

In the future, we plan to integrate our technique with various FPGA‐based implementations of specialized lightweight neural network architectures. This will allow more efficient hardware implementation and leave room for more sophisticated artificial intelligence tasks.

## METHOD

In our proposed method, we aim to reduce the computational complexity of MAC operations by reducing the number of required partial sums. This is done by converting the weight parameters of the pretrained model to approximated MSD representation and designing custom MAC units to perform multiplications with the MSD numbers. Then the overall CNN is implemented with high‐level synthesis, containing the custom MAC units as their building blocks.

### MSD approximation of weights

Minimum signed digit (MSD) [23] representation uses the values {−1, 0, +1} to represent a binary number. The MSD recoding algorithm always results in a minimum number of nonzero digits for a given binary number. The conversion of an unsigned binary number to an MSD representation can be performed using the lookup table in Tab.5. Because of its characteristics, an eight‐digit MSD number can have a maximum of four nonzero digits. This is equivalent to four partial sums, *i.e*., four shift‐and‐add operations. To reduce the partial sums even further, the number of most significant nonzero bits was restricted. In our study, we implemented the FER model with 3, 2, and 1 nonzero bit ( *p*).

**Figure 2:**
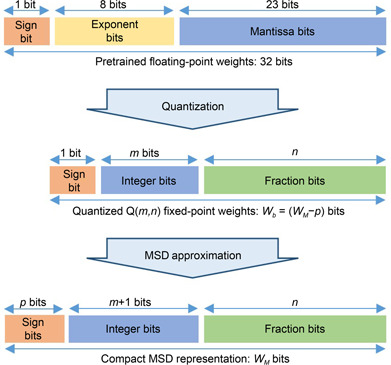
The weight approximation procedure.

Since MSD is a ternary number system, each digit of MSD requires at least two bits to represent the possible states. To address this issue, we used a compact representation to store the MSD weights, as shown in Fig.2. The most significant bits contain the *p* number of sign bits for *p* nonzero digits. At first, we selected the desired bit‐width for the MSD representation ( *W*_*M*_) as 16 bits. From there the bit‐width for the required binary representation ( *W*_*b*_) was deduced by subtracting *p* from *W*_*M*_. So, for different levels of approximation, the quantitation of floating‐point numbers was performed separately.

**Table 5:** Binary to MSD Conversion

Binary representation				MSD representation	
*x*_*i*_	*x*_*i*+1_	*x*_*i*+2_	*c*_*i*_	*y*_*i*_	*c*_*i*+1_
0	0	×	0	0	0
0	1	0	0	0	0
0	1	1	0	1	1
1	0	×	0	1	0
0	1	×	1	$\overset{-}{1}$	1
1	0	0	1	$\overset{-}{1}$	0
1	0	1	1	0	1
1	1	×	1	0	1

*x*_*i*_ and *y*_*i*_ represent the ith digit of binary and MSD representation respectively. *c*_*i*_ represents the carry at the *i*th position in binary representation.

Once we have determined the values of *W*_*M*_ and *W*_*b*_, the floating‐point weights were quantized to Q( *m*, *n*) fixed‐point format, where *m* and *n* represent the number of integer bits and fraction bits, respectively. Then the MSD encoding algorithm in Tab.5 was applied to the Q‐format number from left to right. Once the *p* number of nonzero bits was reached, the rest of the less significant bits were assumed to be zero. All the weights in the convolution layers and the fully connected layers were approximated to the proposed MSD representation. The bias values and other parameters in the normalization layers were kept as Q‐format fixed‐point binary numbers.

### Custom MAC design

**Figure 3:**
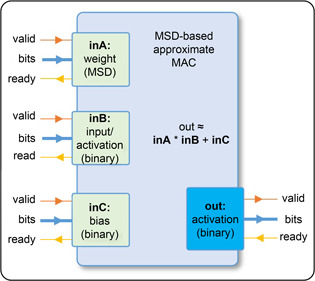
An abstract model of the custom MAC unit.

For inference, since the weight parameters remain unchanged, they can be approximated before synthesis on FPGA. However, the input data and neuron activations are kept as Q‐format binary numbers. Therefore, the custom MAC unit takes the multiplier as a fixed weight value (in approximated MSD form) and the multiplicand as input/activation value (in binary form) to produce a binary Q‐format result, which is forwarded to the next MAC unit as activation value. If a bias value is present, it is stored and added to the result as a binary number. An abstract model of the custom MAC is shown in Fig.3.

Since this is a specialized multiplier, it can neither be used for general‐purpose computation nor can it be implemented by the FPGA’s onboard DSP units.

### FPGA implementation

The MobileNet‐v2 and the ResNet18 model were initially trained on the FER2013 dataset using the Pytorch library. The ResNet18 model was only used in simulation to analyze the model accuracy on different approximation levels of weights. The pretrained MobileNet‐v2 models with approximated weights were implemented on the Altera DE4 development board using the Quartus Prime Pro software package. The custom multiplier was designed as a module with C++ code and then was integrated into the high‐level synthesis library to implement the CNN model. To avoid unexpected data overflow from the multiplication operation, the custom MAC unit was programmed to saturate the result. The oversaturation can also be avoided by carefully optimizing the parameters of the batch normalization layers.

The approximated MSD weights for convolution and fully connected layers are stored in the on‐chip memory, whereas the rest of the binary parameters are in the onboard SSRAM. The custom MAC unit was utilized in convolution operation and for the activation computation of fully connected layers. As for all the rest of the layers, such as batch normalization and average pooling, general‐purpose multipliers or dividers were used.

## COMPLIANCE WITH ETHICS GUIDELINES

The authors Kh Shahriya Zaman and Md Mamun Bin Ibne Reaz declare that they have no conflict of interest or financial conflicts to disclose.

This article does not contain any studies with human or animal materials performed by any of the authors.
